# Analytical reference framework to analyze non-COVID-19 events

**DOI:** 10.1186/s12963-023-00316-8

**Published:** 2023-10-21

**Authors:** María del Pilar Villamil, Nubia Velasco, David Barrera, Andrés Segura-Tinoco, Oscar Bernal, José Tiberio Hernández

**Affiliations:** 1 Department of Systems and Computing Engineering, Universidad de Los Andes, Bogotá, Colombia; 2 School of Management, Universidad de los Andes, Bogotá, Colombia; 3 Departamento de Ingeniería Industrial, Pontificia Universidad Javeriana, Bogotá, Colombia; 4 Escuela Politécnica Superior, Universidad Autónoma de Madrid, Madrid, Spain; 5 School of Government, Universidad de los Andes, Bogotá, Colombia

**Keywords:** Forecasting models, No COVID-19 events, Tuberculosis, Suicide attempt, SARIMA

## Abstract

**Background:**

The COVID-19 pandemic has disrupted the healthcare system, leading to delays in detection of other non-COVID-19 diseases. This paper presents ANE Framework (Analytics for Non-COVID-19 Events), a reliable and user-friendly analytical forecasting framework designed to predict the number of patients with non-COVID-19 diseases. Prior to 2020, there were analytical models focused on specific illnesses and contexts. Then, most models have focused on understanding COVID-19 behavior. There is a lack of analytical frameworks that enable disease forecasting for non-COVID-19 diseases.

**Methods:**

The ANE Framework utilizes time series analysis to generate forecasting models. The framework leverages daily data from official government sources and employs SARIMA models to forecast the number of non-COVID-19 cases, such as tuberculosis and suicide attempts.

**Results:**

The framework was tested on five different non-COVID-19 events. The framework performs well across all events, including tuberculosis and suicide attempts, with a Mean Absolute Percentage Error (MAPE) of up to 20% and the consistency remains independent of the behavior of each event. Moreover, a pairwise comparison of averages can lead to over or underestimation of the impact. The disruption caused by the pandemic resulted in a 17% gap (2383 cases) between expected and reported tuberculosis cases, and a 19% gap (2464 cases) for suicide attempts. These gaps varied between 20 and 64% across different cities and regions. The ANE Framework has proven to be reliable for analyzing several diseases and exhibits the flexibility to incorporate new data from various sources. Regular updates and the inclusion of new associated data enhance the framework's effectiveness.

**Conclusions:**

Current pandemic shows the necessity of developing flexible models to be adapted to different illness data. The framework developed proved to be reliable for the different diseases analyzed, presenting enough flexibility to update with new data or even include new data from different databases. To keep updated on the result of the project allows the inclusion of new data associated with it. Similarly, the proposed strategy in the ANE framework allows for improving the quality of the obtained results with news events.

## Background

The third round of a World Health Organization “pulse survey” reveals that substantial disruptions persist over two years into the COVID-19 pandemic, with about 90% of countries still reporting one or more disturbances to essential health services. In 2020, countries reported that, on average, about half of essential health services were disrupted [27], while in the first three months of 2021, they reported progress, with just over one-third of services during 2021 being interrupted [28]. However, these disturbances have not ended and their effects on the quality of life of the populations continue. At the end of 2021, all regions and countries continue to be severely impacted, with little to no improvement since early 2021 [29]. COVID-19 has impacted health provision in multiple programs such as immunization, HIV, TB, etc. Colombia has been among the 10 countries most affected by COVID-19 despite the early measures taken such as lockdown, quarantine, and social distancing, among others.

In this context, a method or strategy to generate forecasts in other non-COVID-19 pathologies would allow contributing to the analysis of the behavior of diseases, and, as consequence, support decisions related to the number and characteristics of the resources necessary to care for patients with some pathologies that are important in the prioritization of health services.

In the literature, several works characterize and predict the behavior of specific diseases, analyzed in a specific context of public health. For instance, tuberculosis in Malaysia, China, and Algeria is studied by Abdullah et al. [1], Mao et al. [17], Liu et al. [15], Liao et al. [14], and Selmane [22], diarrhea in Cuba, Botswana, and China by Coutin Marie [3], Heaney et al. [7] and Fang et al. [6],acute respiratory infection in Russia and Colombia by Purwanto et al. [20] and Jeronimo-Martinez et al. [10] and Kurniasih et al. [12] and Mishra et al. [18] work on infant mortality in Indonesia, China and India, respectively, while Rodrigues et al. [21], Kessler et al. [11] and Sher [24] analyzed suicide attempt. These works used specific methods to forecast a specific disease, others compared methods from ARIMA models to machine learning models (e.g., Random Forest), including methods such as epidemiological compartmental, exponential smoothing, Holt-Winters, and Box-Jenkins. Taking into consideration the works that present general frameworks to forecast a group of diseases [13] propose a prediction system to monitor and predict the trends of infectious diseases in Taiwan. Later, Lutz et al. [16] provide an overview of infectious disease forecasting with applications to public health, and Jain et al. [9] propose a forecasting framework for Indian Diabetes.

The most recent works published in 2020 and 2021 are mainly focused on predicting the COVID-19 behavior, Sharma and Nigam [23], Chaurasia and Pal [2], Doornik et al. [5], Watson et al. [25] and Oshinubi et al. [19] among others. However, few studies evaluate the impact of COVID-19 on other pathologies. To the best of our knowledge, only Sher [24] examine the impact of COVID-19 on suicide rates, but without using an analytical or a qualitative approach.

Although these results are helpful for public health decision-makers in each specific province, or country, and disease, they are not useful in a different geographic or public health context because of the characteristics of the data or because there is not enough information to be applied.

This paper focused on presenting a general framework (name ANE), developed to forecast several health events different from COVID-19, such as tuberculosis, diarrhea, infant mortality, and suicide attempts, considering data reported by the National Health Institute from Colombia. The forecasting models allow us to evaluate the impact of Coronavirus on those events to help the decision-makers and policymakers make decisions related to public health.

## Framework implementation

The framework proposed and implemented in this work, named “**Analytics for Non-COVID-19 Events” (**ANE, from now on), adapts the ASUM-DM (Analytics Solutions Unified Method for Data Mining) [8] methodology to include and handle specific characteristics of health events and their underlying data.

**Fig. 1:**
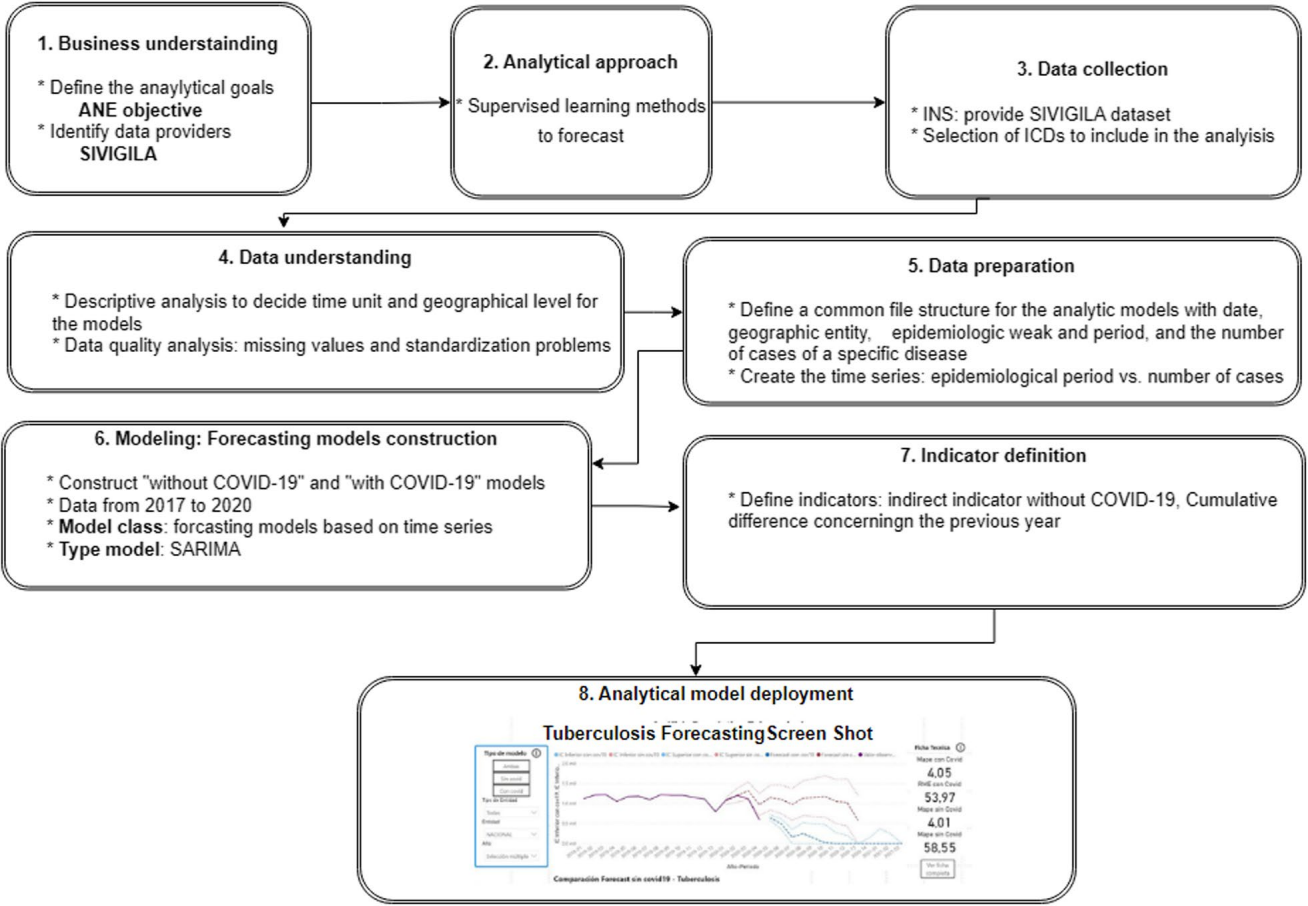
Framework overview

Figure 1 shows the eight consecutive stages that compose the framework, which starts with understanding the business, and goes through collecting and processing the input data, building the predictive models, and the feedback and visualization of the results, among other tasks. The description of each of the stages of this framework and its implementation (for our case study) are presented below in the following subsections (Data Analysis, Model Generation, and Visual Analytics).

### Data analysis

According to the **Business understanding** stage**,** the main purpose of this project is to provide a framework that aims to exploit data related to the number of cases reported in Colombia for specific non-COVID-19 health events to measure the impact of Coronavirus on such events. From the initial list of 4 events, we select tuberculosis and suicide attempts for our case study, following the recommendation of different actors in the sector. For these events, the data were collected from the National Public Health Surveillance System (SIVIGILA^1^—from its acronym in Spanish).

The **Analytical approach** stage consists of reviewing and selecting (from the literature) the set of techniques that will be used to address the objective of this project, i.e., selecting the supervised learning algorithms (specifically, linear regressors and time series methods) that will be used to forecast the number of cases for a specific disease using historical open data from the Colombian health information system from 2015 to 2020. The information collected from SIVIGILA, during the **Data collection** stage, is registered according to the International Classification of Diseases (ICD) format. For each disease, in collaboration with the National Health Institute (INS—from its acronym in Spanish), the selected codes were ones that allow obtaining the number of cases reported per week from the national level to the municipality level, including departments and their capitals. In cases such as Tuberculosis, the datasets contain different codes related to specific types of tuberculosis, and, the decision was taken using the more frequently event in Colombian people as criteria. For this reason, the “pulmonary tuberculosis” is chosen for the analysis.

Additionally, to compare results among geographical regions, population density and the codification of Colombia’s geographic division were taken from the National Administrative Department of Statistics (DANE—from its acronym in Spanish).

In the **Data understanding** stage, the data are inspected for the construction of the analytical models. Using multivariate descriptive analysis and statistical measures (such as mean, standard deviation, minimum, maximum and coefficient of variation), it is possible to define and validate the granularity of the data to be used in the ANE framework, specifically, for the *geographic units* (entities), data at national, departmental and capital level will be used, and for the *time unit*, epidemiological periods will be used (i.e., 4 continuous weeks, having 13 epidemiological periods per year), instead of months. Finally, only data from 2017 to 2020 (which are statistically relevant for the analysis) are used. These decisions were validated by a multidisciplinary team (physicians, engineers, managers, among others).

### Model generation

**Fig. 2:**
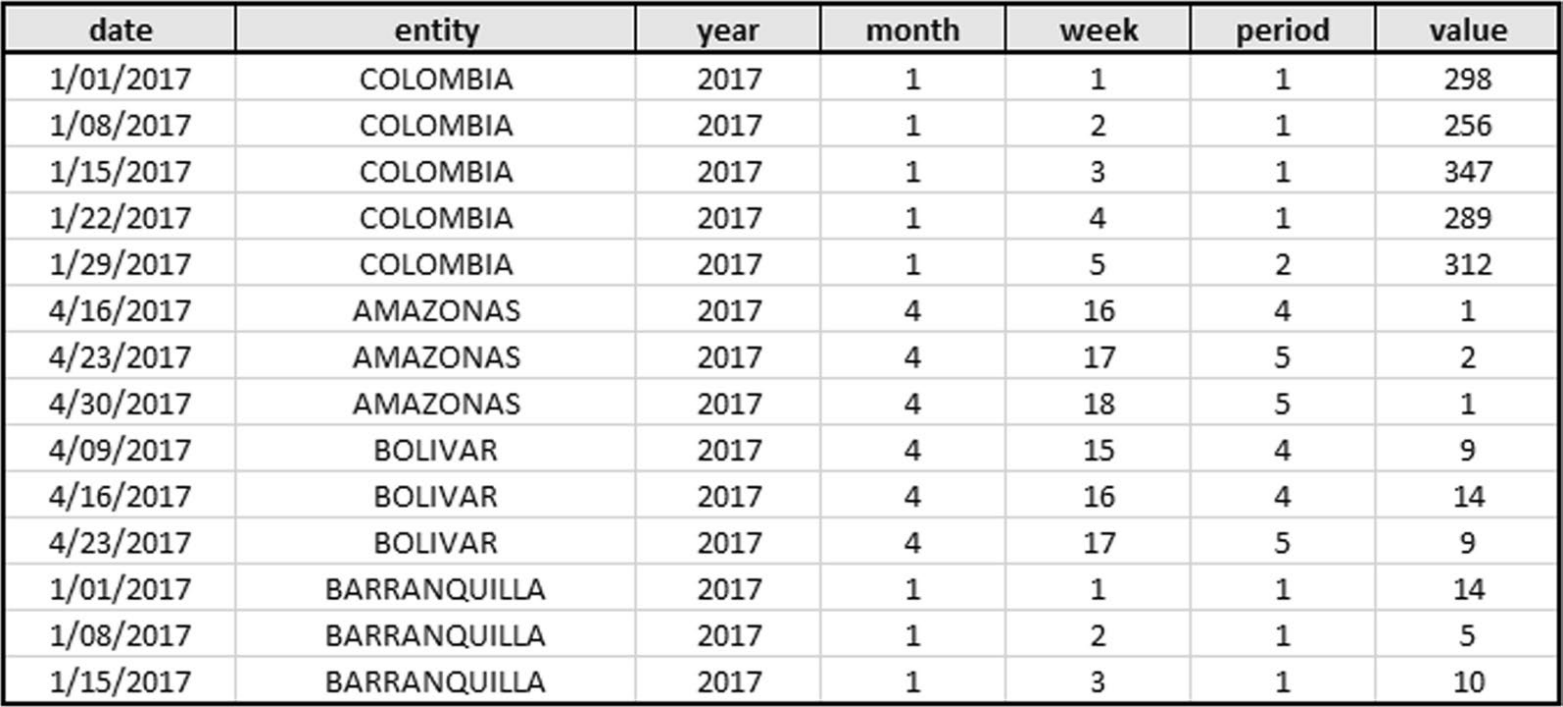
File structure of event data about the national, department, and capital behavior for tuberculosis

With this in mind, during the **Data preparation** stage, a common file structure was used as input for the forecast models. Figure 2 shows the file structure using tuberculosis data as an example. The file is organized in seven columns: date, entity, year, month, week, period (epidemiological period), and value. Entities correspond to a department or capital, and value corresponds to the number of reported cases for each entity. Regarding the analysis of data quality, missing values, duplicate values, and standardization problems were evidenced, which expose some inconsistencies present in the weekly records of several municipalities. Duplicated data were not taken into account (they were removed), and missing data (less than 5%) for a geographic unit were completed with the median of the variable among the time units with data. On the other hand, the problem of data standardization is mostly due to inconsistencies in the spelling of the same geographic unit; for example, "Narino" instead of "Nariño". To avoid this situation, the administrative division of Colombia code (**Divipola**) provided by DANE is used as the entity key.

The **Modeling** stage includes the model selection and the analytic model construction. The main objective in this stage is to create a model to accurately estimate the number of cases ***v*** of a specific event ***x*** for ***n*** periods. The equation is given below:

1 $$v = forecast\left( {x,n} \right)$$

The model created allows us to determine the value of *v* that would have occurred if the COVID-19 pandemic had not happened (referred to as “**without COVID-19”**), including data up to 2019, and predicting the 2020 periods.

We tested three methods to **select the model class**: the polynomial regressions (PR), exponential smoothing (ES) with the Holt-Winters (or triple exponential smoothing) method, and the SARIMA method (the last 2 methods were selected according to the literature recommendations). The PR method served as the baseline model because of its simplicity and its capability to reproduce the trend of the data. The Holt-Winters method is a time series forecasting method that considers the trend and seasonality of the series. The SARIMA model belongs to the Box–Jenkis family and is based on ARIMA (AutoRegressive Integrated Moving Average) models, which use the correlation between data, and like the Holt-Winters method, includes a seasonal component.

Thus, the approach used to construct the forecasting models is based on time series, according to the data characteristics and literature recommendations (in fact, the SARIMA model evidences the best results in the literature review). For all types of models, compliance with the assumptions related to the behavior and nature of the data series over time was validated by statistical tests.

The type of model was selected using the tuberculosis data and evaluating with *mean absolute percentage error* (MAPE) and *tracking signal measure*, which is used as the number of periods that the forecast is between ± 3.2 standard deviations. For each method, the hyperparameters were adjusted using a minimum threshold of 4.0 for the MAPE measure (to avoid the overfitting of the models), and a minimum of 1 available tolerance period for the tracking signal. The Holt-Winters method, and specifically the triple exponential smoothing method, requires that the *alpha, beta, gamma, phi, trend, damped, seasonal, seasonal periods*, and *boxcox* hyperparameters be adjusted. The SARIMA models, on the other hand, have seven hyperparameters (*p, d, q, P, D, Q,* and *m*) that must also be adjusted to obtain the best model. The hyperparameter fitting in the Holt-Winters model and the SARIMA model uses an exhaustive search that maximizes the performance measure (MAPE and the number of periods of the tracking signal). The best results were obtained with the SARIMA approach as shown in Table 1.

**Table 1:** Model comparison

	Time	RMSE	MAPE	AIC	BIC
Base line	18	82	82	82	82
SARIMA	51	71	69	54	54
Holt-Winters	75	70	68	72	72

The **analytical model construction** step consists of selecting the best predictive model for each of the (65 different) geographic units of each event, using the method that provided the best results in the previous step. The selection is performed using a greedy approach in a finite search space of hyperparameters available for the **SARIMA** algorithm. With respect to the model training step, different size distributions were tested for the training and testing datasets (70%-30%, 80%-20%, and 90%-10%). After analyzing the results of the models generated with the experts, we decided to use the 80%-20% size distribution, since it was not only the one that offered the best results, but also the distribution that avoided overestimating and underestimating the absolute percentage error in the predictions. Subsequently, for each model created, the Root Mean Square Error (**RMSE**) and Mean Absolute Percentage Error (**MAPE**) are calculated, and the one with the lowest MAPE and RMSE is selected. In case of ambiguity, the AIC and BIC coefficients are used to select the best model.

### Visual analytics

The objective of the **Indicator definition** stage is to define indicators to quantify the impact of COVID-19 on other health events. The underlying assumption is that, without the measures taken to control the COVID-19 pandemic, it would be reasonable to expect the number of reported cases to be close to the forecast. Then, according to the report on excess mortality presented by DANE [4], and the studied events, two types of impact indicators are proposed. First, for a given period *t*, we analyze the difference between the forecast without COVID-19 (${v}_{t}^{\mathrm{woCOVID}}$) and the reported cases (${O}_{t}$). This is called **“indirect impact**” and it is expressed as a percentage (%) (see Eq. 2). Second, as the sanitary emergency evolved over the year, it was desirable to understand the **“cumulative impact”** from January to a given period *t*. This is estimated as the difference between the cumulative values of the forecast and the reported cases (see Eq. 3).

2 $${\mathrm{Indirect}}_{t}: \frac{ {v}_{t}^{\mathrm{woCOVID}}-{O}_{t} }{{v}_{t}^{\mathrm{woCOVID}}}$$

3 $${\mathrm{Cumulative}}_{t}=\left(\sum_{i=1}^{t}{v}_{i}^{\mathrm{woCOVID}}-\sum_{i=1}^{t}{O}_{i}\right) / \sum_{i=1}^{t}{v}_{i}^{\mathrm{woCOVID}}$$

At the level of the **Analytical model deployment** stage**,** an interactive tool, implemented in PowerBI, is used for the stakeholder’s interaction. This tool is published on Alianza Caoba's public-policy web page.^2^ The tool is composed of three types of pages for each health event. The first one presents the descriptive analysis, the second one the forecast results and the third one the impact indicators. In these three pages offered by the tool, the decision-maker user can filter the information of interest with respect to time and geographic units, and thus, focus only on a specific section of an event to understand its behavior, with the objective of improving his/her decisions. Figs. 11, 12 in Appendix 1, show screen shots of the interactive visualization tool of the prediction for tuberculosis at the national level with/without COVID-19 scenarios. There you can see the results of the prediction models with and without pandemic data, reflecting the estimated impact of each health event in each of the 32 departments and 32 capitals.

## Results

The ANE framework was validated to generate forecasting results for tuberculosis, suicide attempts, diarrhea, diabetes, and infant mortality. This validation allowed us to decide on SARIMA as the forecasting model. In the following paragraphs, a description related to tuberculosis and suicide attempts results is presented. These two diseases are chosen to show the results because they have different behaviors and, respectively, represent two groups: first, diseases that can be confused with COVID-19 and at the same time, due to the lockdown proposed by the government, the number of cases in the population can be reduced; and second, mental diseases than can affect a large number of persons due to social and economic crisis, derived from the COVID-19 pandemic.

### Event 1. Tuberculosis

**Fig. 3:**
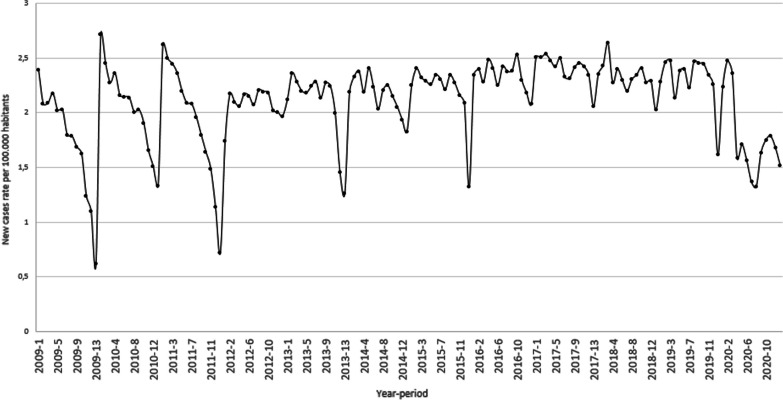
Reported new cases of tuberculosis per 100,000 habitants

Figure 3 presents the number of reported new cases of tuberculosis nationwide, expressed as a rate per 100,000 habitants from 2009 to 2019. This rate has become more stable since 2015 with values ranging from 2.0 to 2.5. Between 2015 and 2019, the average rate per period is 2.3 with a coefficient of variation of 0.19. This value is slightly higher than the one reported between 2012 and 2014 (with an average of 2.1 and a coefficient of variation of 0.23). There is also a systematic decrease in the reported number of new cases in the 13th period of every year. Data presented in Fig. 3 highlights the need to use a forecast model to quantify the number of expected cases and, in this way, the impact of the COVID-19 emergency.

**Fig. 4:**
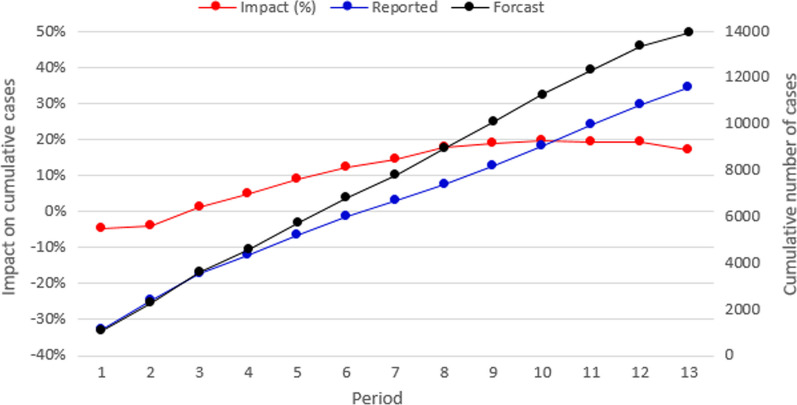
Nationwide cumulative impact on tuberculosis cases per period

**Fig. 5:**
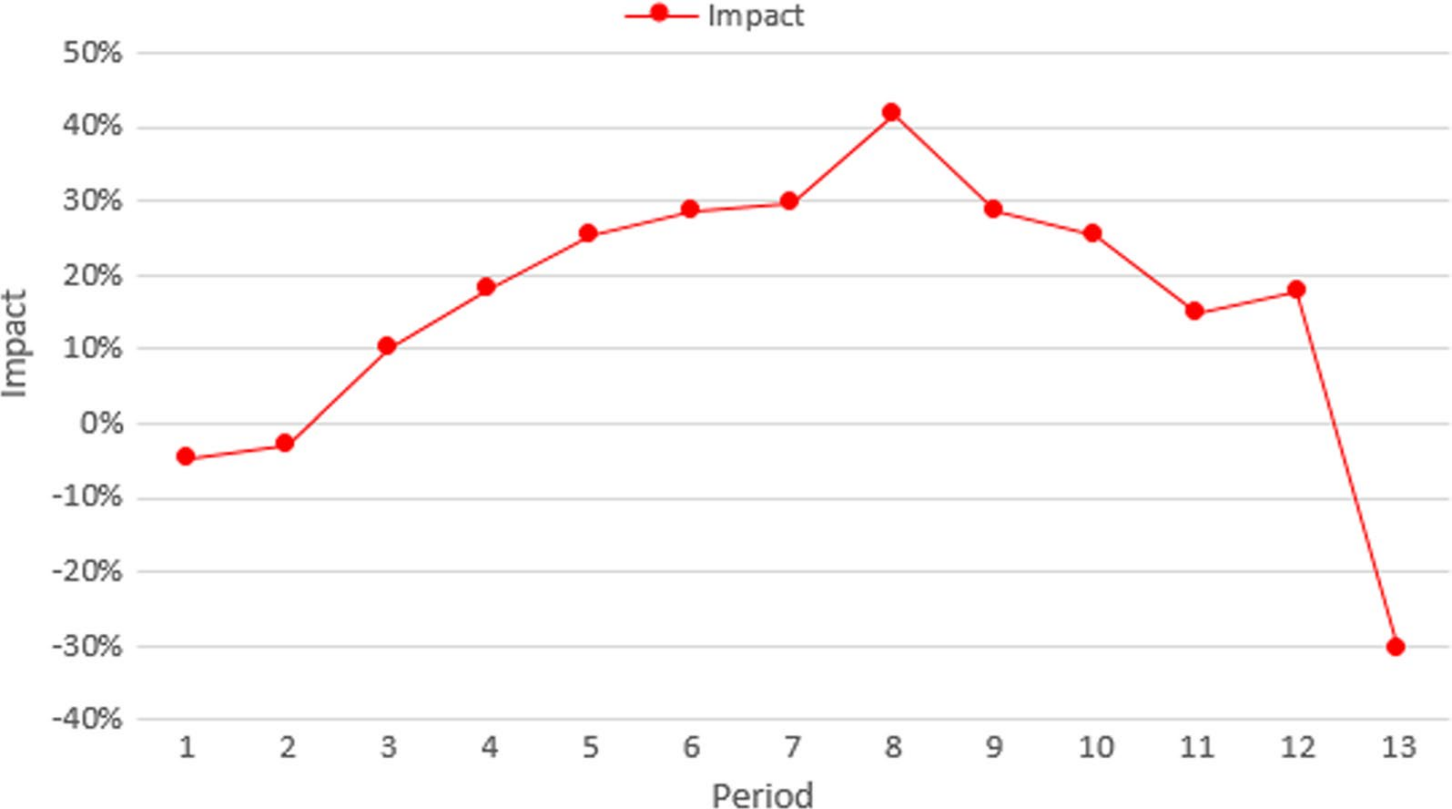
Nationwide indirect impact on tuberculosis cases per period

Figure 4 summarizes the nationwide cumulative impact of the COVID-19 sanitary emergency on the number of tuberculosis-reported cases for each epidemiological period. The black and blue lines present the cumulative forecasted and the reported cases, respectively. In this figure, 13,954 cases were expected to be identified during 2020. However, according to the information retrieved from the epidemiological surveillance information system, only 11,571 were reported. This means that around 17% of the cases (2383) were not identified. The red line in Fig. 5 presents the difference between the two cumulative values, for each epidemiological period. In Colombia, social distance restrictions were adopted for the first time during the fourth epidemiological period. From this time, the cumulative impact of the emergency on the number of identified cases ranges between 5 and 20%. However, when analyzed by period, the impact varies from 10 to 40%. In Fig. 3, during the eighth period, the number of reported cases was 40% lower than the forecast, however, in the final period of the year, the report was 30% higher than expected.

**Fig. 6:**
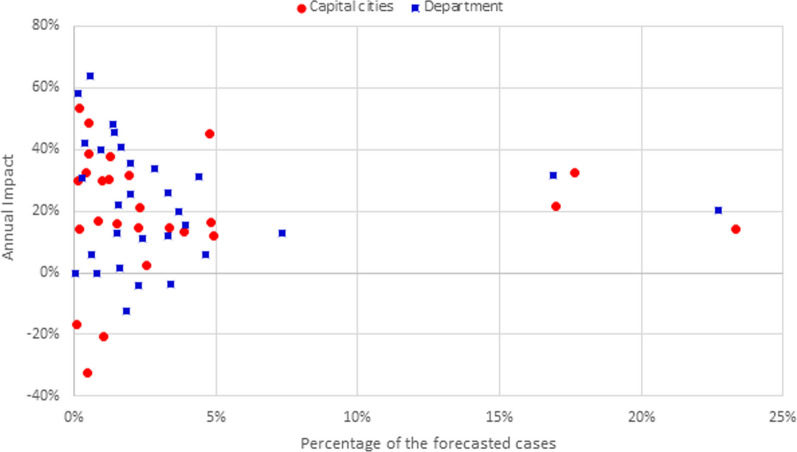
Annual impact on tuberculosis for each capital and department, and its participation in the total number of forecasted cases

Figure 6 presents the observed impact for each department and main municipality and its participation in the total number of forecasted cases. The participation was calculated independently for municipalities and departments, and the impact was quantified with the cumulative values. Consequently, the red point (23%, 14%) represents a municipality accounting for 23% of the forecasted cases (for all capital cities) in which the total number of identified cases (during 2020) is 14% lower than the forecasted value. Similarly, the blue square (23%, 20%) represents a department with 23% of the forecasted cases and an impact of 20%. In the same figure, most departments and municipalities (81% and 91%, respectively) account for less than 5% of the total number of forecasted cases. These can be classified into three groups according to the cumulative impact. For the first group, no additional action is required, as the variation between 2019 and 2020 can be considered normal. In 34% of the municipalities and 37% of the departments, the impact ranges from 0 to 20%. Additionally, in three departments and two capitals, the impact is negative and greater than − 20%, which means that more cases were reported than forecasted. However, there is a second group with impacts varying between 20 and 64%. For these departments and capitals, there is a risk that some of the new cases were not identified due to the disruptions in the health system. Lastly, two municipalities reported 21% and 32% cases more than forecasted. It is important to alert local health authorities of a possibly atypical increase in the new tuberculosis cases.

### Event 2. Suicide attempts

**Fig. 7:**
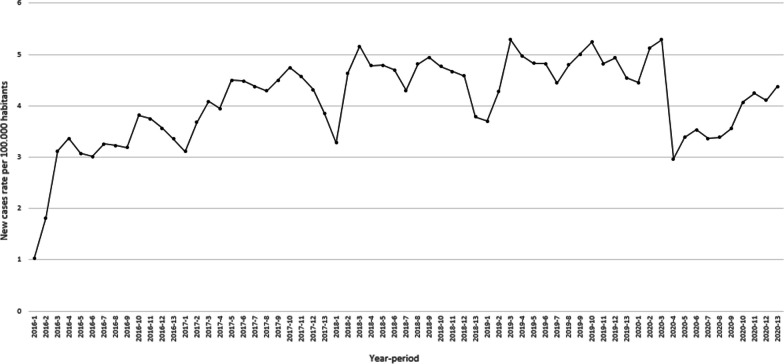
Reported new cases of suicide attempts per 100,000 habitants

Figure 7 presents the reported number of suicide attempts, expressed as a rate per 100,000 habitants during the period 2016–2019. The graphic presents a rising trend in the rate between 2017 and 2019. While in 2017, the average rate per month was 4.2 (coefficient of variation of 0.11), in 2019, the average rate increased up to 4.8 and the coefficient of variation decreased to 0.09.

**Fig. 8:**
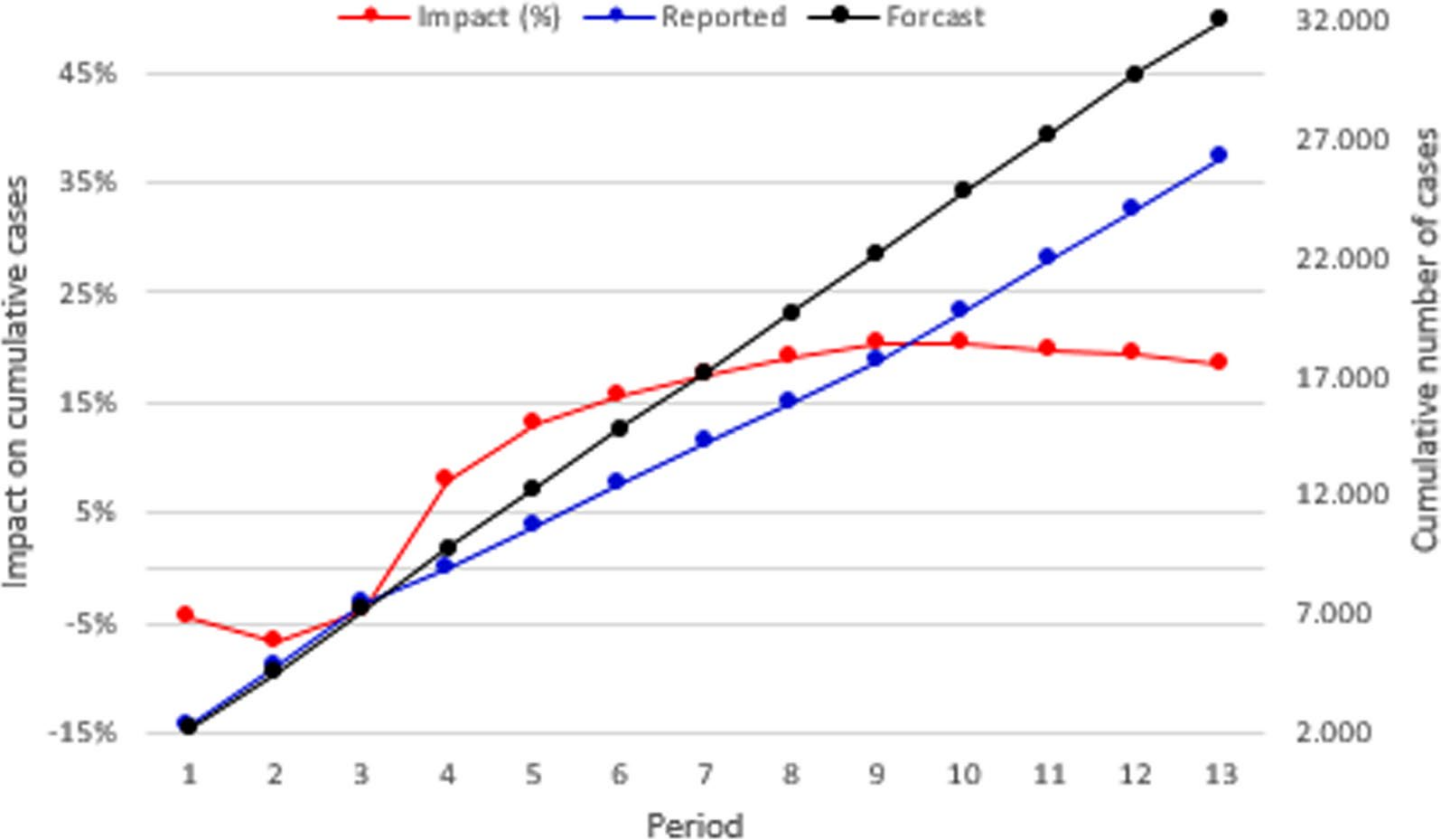
Nationwide cumulative impact on suicide attempts per epidemiological period during 2020

**Fig. 9:**
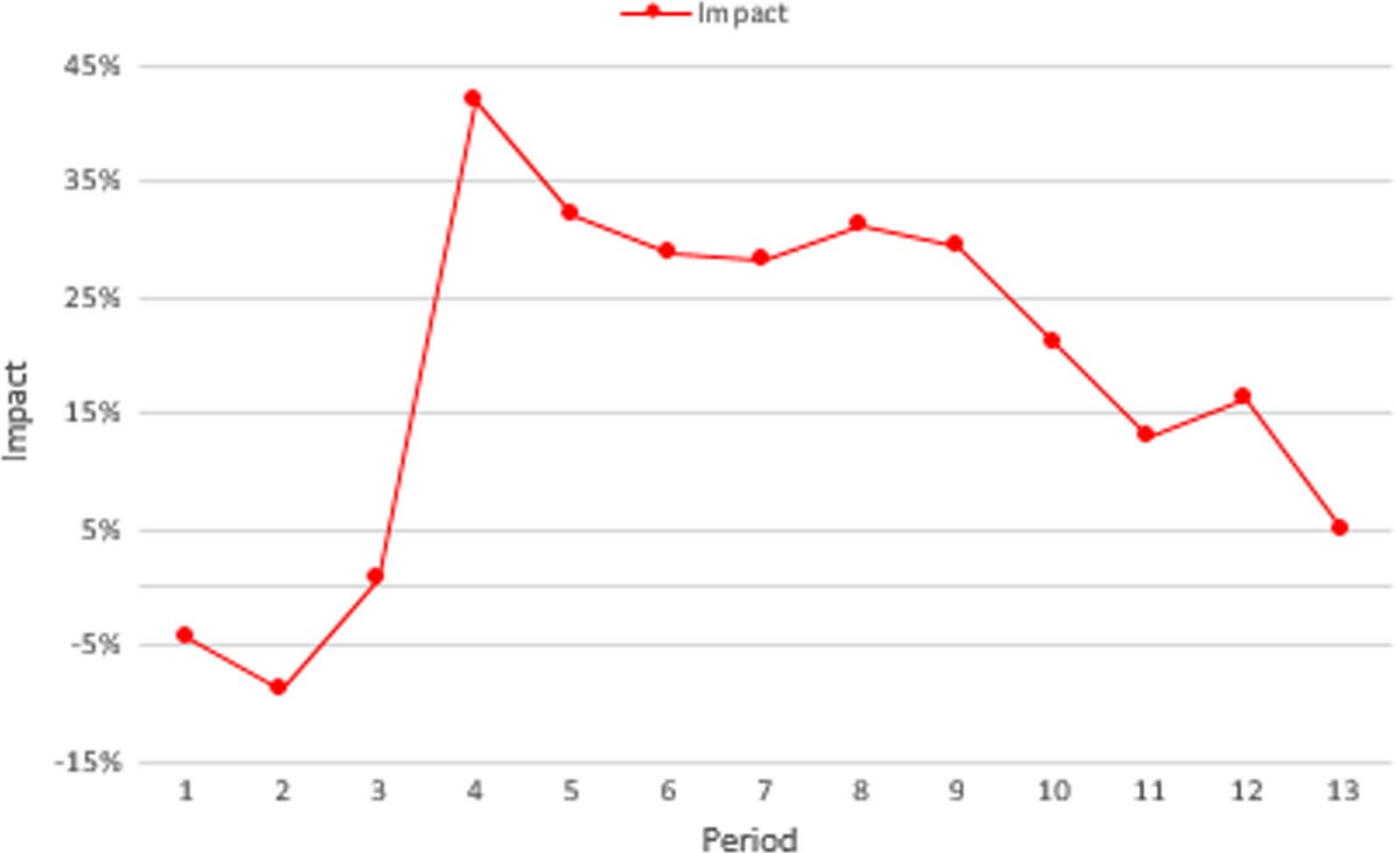
Nationwide indirect impact on suicide attempts per epidemiological period during 2020

Figure 8 summarizes the nationwide cumulative impact of the COVID-19 sanitary emergency on the reported number of suicide attempts for each epidemiological period. Can be notice that 32,029 cases were expected (black line) to be reported over 2020. However, according to information retrieved from the surveillance information system, only 26,143 were registered (blue line). While an average rate of 2464 (coefficient of variation of 0.05) new cases per period was expected, between the fourth and the ninth epidemiological periods less than 1800 cases were registered (an average of 1697 cases per period). The red line in Fig. 8 presents the difference between the two cumulative values for each epidemiological period. From the adoption of the social distance restrictions, the cumulative impact of the emergency on the number of reported cases ranges between 8 and 20%. However, when analyzed by period, the impact varies from − 9 to 42%. Figure 9 shows that during the fourth period, the number of reported cases was 42% lower than the forecast. Additionally, in the final period of the year, the report was 18% higher than expected.

**Fig. 10:**
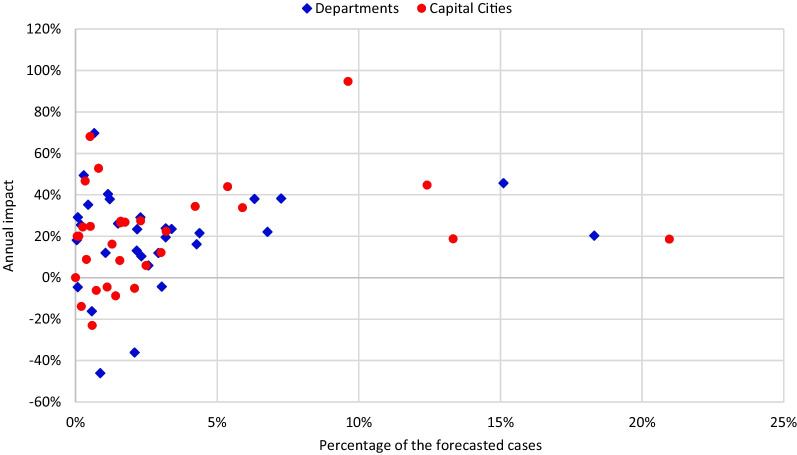
Annual impact on suicide attempts for each capital and department and its participation in the total number of forecasted cases

**Fig. 11:**
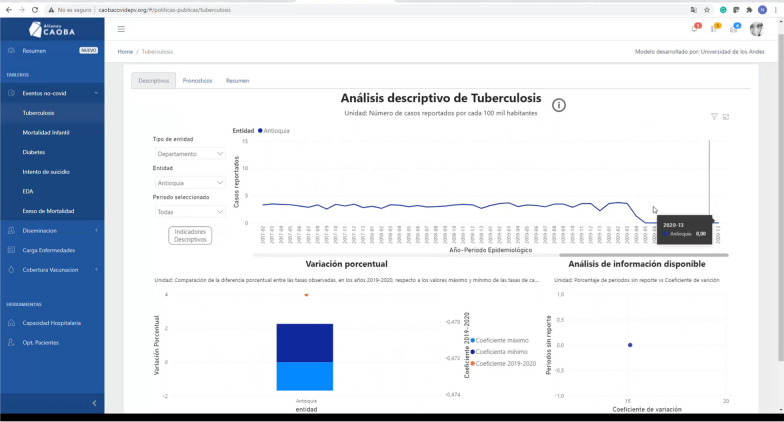
Interactive visualization tool developed—Screen shot of Descriptive analysis page—Tuberculosis case

**Fig. 12:**
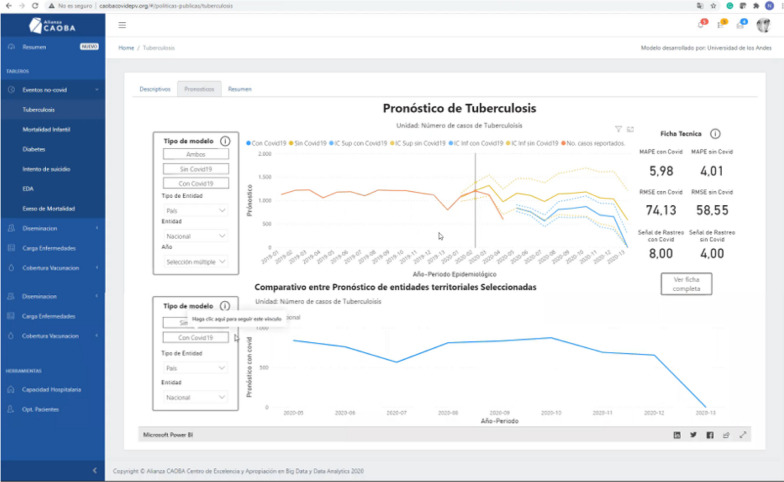
Interactive visualization tool developed—Screen shot of Predictive analysis page—Tuberculosis case

Figure 10 presents the observed impact of each department and municipality and its participation in the total number of forecasted cases. The participation was calculated independently for municipalities and departments, and the impact was quantified with the cumulative values. Consequently, the red point (21%, 19%) represents a municipality accounting for 21% of the forecasted cases (for all capital cities) in which the total number of identified cases (over 2020) is 19% lower than the forecasted value. Similarly, the blue square (18%, 20%) represents a department with 18% of the forecasted cases and an impact of 20%. From the same figure, it is visible that five departments represent 54% of the forecasted cases with impact ranging from 20 to 46%. Similarly, four municipalities represent 56% of the forecasted cases. The impact in three out of these four cities varies between 19 and 45%. However, one municipality accounts for 10% of the forecasted cases, and only 5% of these cases were reported (impact of 95%). Additionally, in five departments and six municipalities, the number of reported cases was greater than expected, leading to a negative impact indicator. For these departments, the impact ranges between − 46 and − 5%, and for the capital cities it changes from -23% to -5%. Lastly, while the most common range for impact among the departments is between 20 and 30% (45% of the departments), in 23% of the capital cities the impact ranges from 0 to 20%.

## Discussion

The discussion is organized in two parts: (1) related to framework validation challenges in Colombia context and (2) related to tuberculosis and suicide attempts.

### Framework validation challenges

During the pandemic period, the majority of resources were focused on COVID-19. In this way, it was not possible to obtain time series about other events with detailed data, including for example, sex, age, socioeconomic level, occupation, features about location (rural or urban), type of employment, nutritional status, and type of affiliation at the health system, to have clustering analysis and create descriptive and forecasting models for each one of these groups, adding more information and enabling other kinds of analytics models. The solution was to use open data about different events and provide a common way to report data to facilitate the preprocessing and model generation. Although, we can create and validate the framework with this information, it was necessary to propose a minimalist data strategy based only on time series with the number of cases by event and by geography and try to tune the forecasting models to obtain, in general, results with high quality. These restrictions affect the analysis of results because cities or departments with similar characteristics at external aspects not included in models had quality metrics of forecasting models that were very different, and it was not possible to balance and change these situations to improve quality. The use of open data avoided the analysis of events such as maternal mortality because the recollection process was changed during the period of analysis and the data was not comparable. These issues generate challenges and opportunities to improve the quality, opportunity, and level of detail of the data shared as open data, complying with ethical and privacy aspects.

### Tuberculosis and suicide attempts

The main findings of this study showed that around 17% of the tuberculosis cases (2383) were not identified, due to the disruptions in the healthcare system by COVID-19. This interference especially affected some cities and regions with impacts varying between 20 and 64%. For those departments and municipalities, there is a risk that some of the new cases were not identified with a higher number of untreated TB patients and increased risk of TB transmission at the community level.

Colombia and other countries can use these estimations to be prepared of increasing TB patients post-pandemic due to late detection and increased community transmission. The traditional TB surveillance system based on historically reported cases needs to be adapted considering the changes in access, report, and transmission. The new surveillance system proposed by WHO in 2022 aims to facilitate the implementation of digital, case-based, real-time surveillance systems for TB.^3^ This new surveillance system can be favored by the modeling used in this study.

In the WHO second round of the National pulse survey on continuity of essential health services during the COVID-19 pandemic, over 40% of countries reported interferences that affect the availability of, and access to, quality services, including for the most vulnerable individuals. 51% of countries reported disruption of TB diagnoses and treatment, and 44% of countries reported disruptions in suicide prevention programs [27].

The WHO TB goals included a key milestone of a 75% reduction in tuberculosis deaths by 2025, compared with 2015, and a decline in global tuberculosis incidence rates to 10% per year by 2025 [26]. The strategy to accomplish this milestone is based on early diagnostics, treatment, and reduction of community transmission. However, the TB incidence reduction in 2020 could be explained because a descent in the TB laboratory tests and in the number of patients starting treatment, during lockdown periods. So, there is a need to analyze differently this reduction and monitoring the risk of an increased number of cases during the following years, due to the missing cases who are in the community increasing transmission.

This study has some limitations due to the late access of the datasets and interruption of health facilities reports due to COVID-19. Additionally, it is important to note that as the information, in the case of TB, started to be recorded in 2009, the first three years might have quality problems and it is not possible to quantify the effect of the set-up period. In a similar way, the information of suicide attempts, started to recorded in 2016 and it is also sensible to have quality problems. In both datasets there is a systematic decrease in the reported number of new cases in the 13th period of every year, rather than a behavior of the disease, this seasonality reflects maybe the hiring cycle of the people in charge of this task. Perhaps more importantly, the data presented in Figs. 3 and 7 highlights the need to use a forecast model to quantify the impact of the COVID-19 emergency. In this way, using a pairwise comparison among the average number of cases could lead to an over (or under) estimation of the impact.

## Conclusions

This paper presents ANE, a framework to analyze and evaluate the effect of COVID-19 related health events other than coronavirus disease using open data provided by different sources. The framework provides a strategy to construct forecasting models based on time series (specifically, the SARIMA method) and defines indicators to guide decision-making processes. The framework includes data analysis, model generation, definition of some specific indicators and, in general, the deployment phase for visual analysis purposes. Furthermore, two indirect impact indicators are included in the proposal, and despite the results not being conclusive, due to the lack of information, they are easy to estimate and interpret.

The forecasts showed a gap between expected and reported TB cases and demand a validation of different actors including SIVIGILA at the level of data and forecasting models' quality. It is possible that according to these results, a public health intervention to detect these potential TB cases in the community, because undetected cases might lead to an increase in community transmission. Active screening and recovering TB diagnostic capacity are key elements in the reduction of this gap.

After implementing the ANE framework in our case study, it proves to be flexible and adaptable. Flexible, because the stages are not straitjackets to which the same amount of time and resources must always be dedicated, and adaptable, because it can be applied not only to the health events presented in this study (tuberculosis and attempted suicide), but also to other health events, such as diarrhea, infant mortality, and diabetes, among others.

Although each analyzed health event has different behavior and its characteristics include different data quality levels, the ANE framework proposes a single strategy to develop forecasting models that allow to include these characteristics, obtaining good results according to error measures and performance indicators (MAPE less than 20% and stable tracking signals greater than a forecast period).

The models developed on tuberculosis and suicide attempts, use data reported up to December 2020. However, the ANE framework allows the inclusion of new data associated with those events, allowing to update the result of the project. Similarly, it is possible to use the ANE framework to include new events, following the proposed strategy, to be made at each stage, to improve the quality of the results obtained. In this way, data about diarrhea, diabetes, and infant mortality were also analyzed using the framework and the results are included on the dashboard application developed. The validation of the framework with distinct health events suggests its adjustment on different contexts, including other countries.

According to the aforementioned issues, the main contributions of this paper focus on four aspects:

- The ANE framework allows analyzing different kinds of Non-COVID-19 diseases, using forecasting models and descriptive analyses.
- The ANE framework uses heterogeneous information available in public datasets.
- The ANE framework includes forecasting and descriptive analyses at different geographical levels: department (32), capital (32), and national (1), contributing to the statistics currently reported.
- The dashboard application was developed to provide an interactive analysis to improve the public health decision-making process. The results of the descriptive analyzes, forecasting models, and indirect impact indicators obtained with the ANE framework are displayed in a dashboard application developed in the context of CAOBA. This application allows to compare analyses at the national, departmental, and capital levels, in epidemiological periods and shows the value of the different indicators implemented, key elements to support decision-makers.

Finally, some opportunities can be exploited for future work. The use of detailed data sources to verify and complement decisions about framework configuration, and to improve the level and completeness of results to support health decision makers. The automation of the collecting and transformation process of the original data sources to a standard format, and a validation process with experts of different institutions related to public health, can improve the potential of this framework and its usability in real contexts of decision-making process.

## Data Availability

A repository link with the data and source code is available at https://ansegura7.github.io/covid19-col-disease-impact/

## Appendix 1: Screen shots of the visual analytic tool developed

See Figs. 11, 12.

## Abbreviations

DANE: Departamento Administrativo Nacional de Estadística (National Administrative Department of Statistics)
INS: Instituto Nacional de Salud (National Health Institute)
MoH: Ministerio de salud y protección social* (*Ministry of health)
WHO: World Health Organization

## Footnotes

1 SIVIGILA (https://www.ins.gov.co/Direcciones/Vigilancia/Paginas/SIVIGILA.aspx), is the Colombia National Public Health Surveillance System. It was created to provide in a systematic and timely way, the monitoring and risk analysis about events of interest in public health in the country.

2 http://caobacovidepv.org/#/politicas-publicas/. This page is the result of a project in the context of Colombian public health, and some links are not available.

3 https://www.who.int/publications/digital/global-tuberculosis-report-2021/featured-topics/case-based-surveillance.
